# Evaluating *Allium sativum* and *Vitis vinifera* Extracts as Potential Adjuvant Agents in Colorectal Cancer: Insights from HT-29 and Caco-2 Models

**DOI:** 10.3390/biomedicines13081968

**Published:** 2025-08-13

**Authors:** Raquel Bodoque-Villar, Elisabet María Roldán-Díaz, Leticia Serrano-Oviedo, Hernán David Garzón-Quintero, Mónica Cañete-Rodríguez, Luis Antonio Gómez, Ignacio Gracia, Juan Francisco Rodríguez, Natalia Bejarano-Ramírez, Gema Verdugo-Moreno, José Ramón Muñoz-Rodríguez, Francisco Javier Redondo-Calvo

**Affiliations:** 1 Translational Research Unit, University General Hospital and Research Institute of Castilla-La Mancha (IDISCAM), 13004 Ciudad Real, Spain; rbodoquev@sescam.jccm.es (R.B.-V.); lserranoo@sescam.jccm.es (L.S.-O.); hgarzon@sescam.jccm.es (H.D.G.-Q.); mcaneterodriguez@sescam.jccm.es (M.C.-R.); gverdugo@sescam.jccm.es (G.V.-M.); 2 Translational Research Group, GAI of Ciudad Real, Spain, Research Institute of Castilla-La Mancha (IDISCAM), 13004 Ciudad Real, Spainignacio.gracia@uclm.es (I.G.); juan.rromero@uclm.es (J.F.R.); 3 Department of Chemical Engineering, Institute of Chemical and Environmental Technology, Universidad de Castilla-La Mancha, 13071 Ciudad Real, Spain; 4 Faculty of Medicine, Universidad de Castilla-La Mancha, 13071 Ciudad Real, Spain; nbejarano@sescam.jccm.es; 5 Department of Pediatrics, University General Hospital, 13004 Ciudad Real, Spain; 6 Department of Anesthesiology and Critical Care Medicine, University General Hospital, 13004 Ciudad Real, Spain

**Keywords:** *Allium sativum*, *Vitis vinifera*, thiosulfinates, oligomeric proanthocyanidins condensed, Caco-2 and HT-29

## Abstract

**Background/Objectives**: Colorectal cancer (CRC) remains a major health challenge due to its high incidence and resistance to conventional therapies. Natural compounds have gained attention as potential adjuvant treatments. The study assesses whether combining *Allium sativum* and *Vitis vinifera* [rich in oligomeric proanthocyanidins condensed (OPCs)] extracts enhances cell viability reduction and migration inhibition. **Methods**: Human colorectal cancer cell lines (Caco-2 and HT-29) were treated with increasing concentrations of both extracts individually and in combination. Cell viability was assessed using MTT assays, while migration was evaluated through scratch wound assays. Synergistic effects were analyzed using Combenefit software. **Results**: Both extracts significantly reduced cell viability in a dose- and time-dependent manner. The combination of both extracts led to an enhanced reduction in cell viability, with a transient synergistic effect observed at 24 h in HT-29 cells. Regarding migration, OPCs showed a transient anti-migratory effect at 6 h in HT-29 cells, but no significant impact was observed in Caco-2 cells or at later time points. **Conclusions**: These findings suggest that *Allium sativum* and *Vitis vinifera* extracts have potential as complementary treatments for colorectal cancer, mainly through their effect on cell viability. This study opens a field of research on the possible therapeutic effects of natural extracts.

## 1. Introduction

Colorectal cancer (CRC) is one of the leading causes of cancer-related mortality worldwide, with increasing incidence rates in both developed and developing countries [1]. Despite advances in treatment strategies, including surgery, chemotherapy, and targeted therapies, CRC remains a significant public health challenge due to its aggressive nature and high recurrence rates [2]. Numerous natural products present in bioactive substances act as chemoprotective agents against various types of cancer, due to their anti-inflammatory, antioxidant, and antiproliferative properties. These substances are found in vegetables, fruits, plant extracts, and herbs, making them promising candidates for interventions in oncology [3].

*Allium sativum* (garlic) is a bulbous plant species widely recognized for its health benefits, particularly its antimicrobial, anti-inflammatory, antioxidant, and anticancer activities [4]. The medicinal properties of garlic have been extensively studied, focusing on its sulfur-containing bioactive compounds (thiosulfinates), such as allicin [5,6]. Generally, the medicinal use of garlic has been based on the properties attributed to allicin in most studies. However, due to the instability of this compound, several researchers have chosen to focus on the properties of other allicin-derived compounds [7,8,9]. Similarly, *Vitis vinifera* (grape) is rich in various polyphenol compounds, including proanthocyanidins (PAs), anthocyanins, and resveratrol. These polyphenols have demonstrated numerous biological activities and exhibit a wide range of biological effects, such as anti-inflammatory, antioxidant, antidiabetic, antitumor, and cardioprotective properties [10,11]. Among PAs, a specific subclass known as oligomeric proanthocyanidins condensed (OPCs) has gained attention due to its potent biological activity. Structurally, OPCs consist of flavan-3-ol monomers linked through interflavan bonds, forming oligomers with varying degrees of polymerization. Notably, OPCs have demonstrated promising chemopreventive effects in cancer models, including the inhibition of cell proliferation, induction of apoptosis, and suppression of metastatic processes. Their stability, bioavailability, and low toxicity profile make them compelling candidates for adjunctive cancer therapies [12,13,14].

In a previous study carried out by our research group, the use of a new thiosulfinate-enriched *Allium sativum* extract, as an adjuvant to chemotherapeutic drugs in the treatment of colorectal tumors, was experimentally examined. In this study, a greater antiproliferative effect was observed after combining garlic extract with first-line chemotherapeutic agents for colorectal cancer [7]. Due to these promising results, we aimed to evaluate whether *Vitis vinifera* extract with high oligomeric proanthocyanidins and oligomeric proanthocyaninds condensed for molecular condensation degrees equal or more than 4 enhances the efficacy of *Allium sativum* extract in the reduction of cell viability and the inhibition of cell migration. Our findings contribute to the growing body of evidence supporting the potential use of natural compounds in cancer therapy and provide insights into the mechanisms underlying their anticancer effects.

## 2. Materials and Methods

### 2.1. Vitis vinifera and Allium sativum Extracts: HPLC Analyses

The polyphenol extract used in this study was obtained from white grape seed according to the following procedure: after crushing the grape seeds, it was subjected to solid−liquid extraction using water as a solvent with pH control until the solid was exhausted. The extract was stabilized with sulfites at a concentration of less than 500 ppm, and it is subjected to a column atomization process under controlled pressure and temperature conditions, obtaining a solid with a water activity of less than 0.3 units, in according to procedures accomplished in AVIALSA, Ctra. Munera (CM-3119) n° 7, 02600 Villarrobledo, Albacete, Spain, as the manufacturer. For the determination of proanthocyanidins in the samples used in this work, a 4.6 × 150 mm Zorbax Sb-aq column with 5 μm particles was used as the stationary phase. The mobile phase was composed of water, methanol, acetonitrile, and formic acid of HPLC quality in gradient with the Socratic model at 28 °C, with an injection volume of 50 microliters and a wavelength set at 280.4 nm. In this way, an extract rich in proanthocyanidins from grapes was obtained with a majority concentration of condensed oligomeric proanthocyanidins with a degree of condensation greater than 4 monomeric units. For HPLC determination, gallic acid, (±)-epicatechin, (±)-catechin, and (±)-epigallocatechin, as well as oligomeric proanthocyanidins A1, A2, B1, B2, C1, and oligomeric proanthocyanidins condensed (OPCs), were used as external standards provided by Analysis Vinicos (Tomelloso, Ciudad Real, Spain).

The *Allium sativum* extracts were Aliben^®^ provided by Alvenpe Salud SL, Plaza de la Reina n° 2, 16660 Las Pedroñeras, Cuenca, Spain. These extracts were obtained from purple garlic from La Mancha under patented conditions (patent number: ES2306597 B1). The thisoulfinate content of Aliben^®^ was determined using a Shimadzu HPLC apparatus (Disburg, Germany) with a methanol/water (50/50) mobile phase and a Supelcosil C18 column (150 4,6 mm; i.d. = 5 μm) stationary phase. Solute detection was carried out using a UV-vis detector set a 254 nm. The mobile phase flow rate was 1 mL/min. Dimethyl sulfoxide (DMSO; Sigma-Aldrich, Saint Louis, MO, USA) was used as the internal standard provided by Analysis Vínicos (Tomelloso, Ciudad Real, Spain).

### 2.2. Cell Lines and Culture Conditions

Caco-2 (ATCC*^®^* HTB-37) and HT-29 (ATCC*^®^* HTB-38) human colorectal adenocarcinoma cell lines were obtained from the American Type Culture Collection. Cells were cultured in Dulbecco’s Modified Eagle Medium (DMEM, Sigma Aldrich, Darmstadt, Germany), supplemented with 10% fetal bovine serum (FBS; Sigma-Aldrich, Burlington, MA, USA), and antibiotics (penicillin 100 IU/mL and streptomycin 100 μg/mL; Sigma Aldrich, Darmstadt, Germany) and maintained at 37 °C in a humidified atmosphere containing 5% CO_2_. Mycoplasma contamination tests were frequently run in our laboratory and the experiments were performed between passages two to ten.

### 2.3. MTT Viability Assay

To study cell viability, 3-[4,5-dimethylthiazol-2-yl]-2,5-diphenyl-2H-tetrazolium bromide (MTT) assay was performed. Briefly, HT-29 was seeded into 24-well plates at a density of 25 × 10^3^ cells/well and Caco-2 was seeded at a density of 3 × 10^4^ cells/well; then, they were allowed to adhere overnight in a humidified incubator at 37 °C and 5% CO_2_. After exposure to extract for 24 h, 48 h, and 72 h time periods, 25 μL of MTT reagent [5 mg/mL in phosphate-buffered saline (PBS), Sigma-Aldrich, Burlington, MA, USA] was added to each well, followed by incubation at 37 °C for 1 h, after which formazan crystals were solubilized by adding 500 μL of DMSO to each well. Plates were placed on a shaking incubator for 10 min and read at 570 nm by a microplate reader (Dynex Spectra MR, Chantilly, VA, USA) using a reference wavelength of 650 nm in triplicate. Cell viability was expressed as a percentage relative to the untreated control and is represented as box plots illustrating the distribution across three independent biological replicates. Drug interaction analysis was performed using Combenefit software (v2.021, Cancer Research UK Cambridge Institute, Cambridge, UK). 

### 2.4. Scratch Wound Assay

Cell migration was assessed using the scratch wound assay. HT-29 and Caco-2 cells were seeded into 6-well plates at a density of 8 × 10^5^ cells/well and cultured at 37 °C and 5% CO_2_ until reaching full confluence. A uniform scratch was created in a monolayer using a sterile 10 μL pipette tip. Detached cells were removed by washing twice with PBS. Cells were then incubated in serum-free DMEM supplemented with treatment compounds as indicated. Images of the wound area were captured at 0, 3, 6, and 24 h using an inverted microscope (Nikon, Japan) at a magnification of 10. Wound closure was quantified using ImageJ software (NIH, Bethesda, MD, USA, version 1.54p, February 2025) by calculating the percentage reduction in the wound area relative to the initial wound width, represented as box plots showing the distribution of three independent experiments.

### 2.5. Statistical Analysis

**Figure 1:**
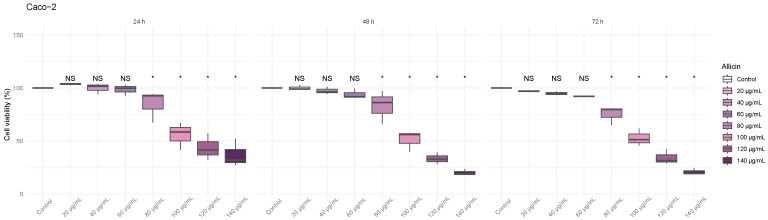
**Effect of various thiosulfinate-enriched *Allium sativum* extract concentrations on cell viability of the colon cancer cell line Caco-2 after 24 h, 48 h, and 72 h incubation.** MTT assays were performed, and the percentage of viable cells was calculated. * *p*-value of < 0.05 vs. control.

**Figure 2:**
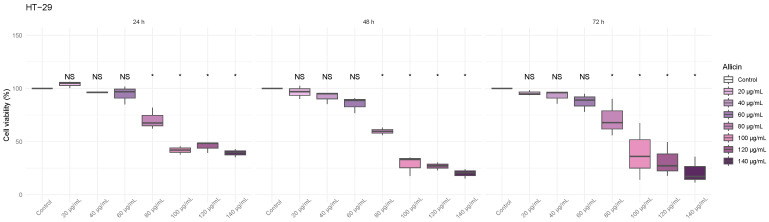
**Effect of various thiosulfinate-enriched *Allium sativum* extract concentrations on cell viability of the colon cancer cell line HT-29 after 24 h, 48 h, and 72 h incubation.** MTT assays were performed, and the percentage of viable cells was calculated. * *p*-value < 0.05 vs. control.

**Figure 3:**
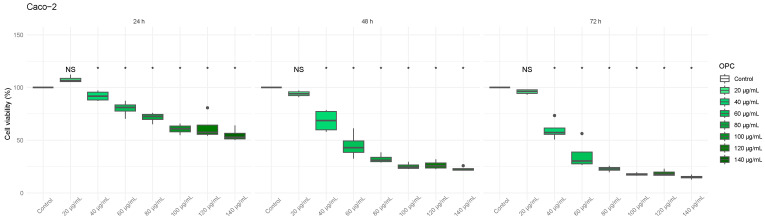
**Effect of various *Vitis vinifera* extract concentrations on cell viability of the colon cancer cell line Caco-2 after 24 h, 48 h, and 72 h incubation.** MTT assays were performed, and the percentage of viable cells was calculated. * *p*-value of < 0.05 vs. control.

**Figure 4:**
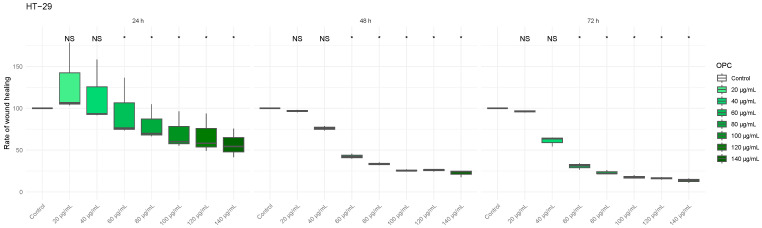
**Effect of various *Vitis vinifera* extract concentrations on cell viability of the colon cancer cell line HT-29 after 24 h, 48 h, and 72 h incubation.** MTT assays were performed, and the percentage of viable cells was calculated. * *p*-value of < 0.05 vs. control.

**Figure 5:**
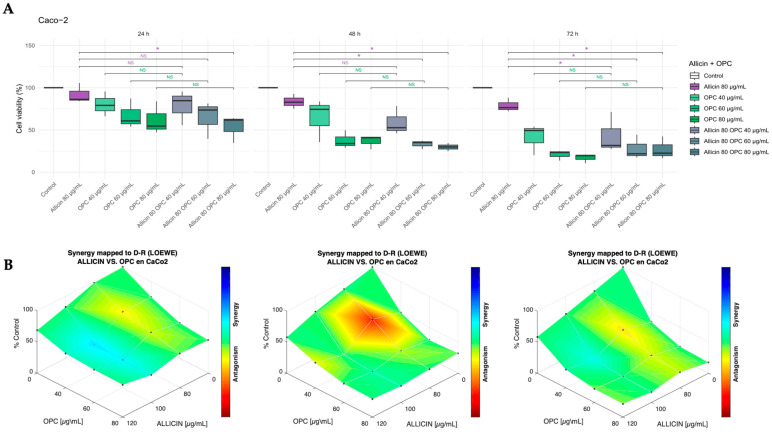
**Effect of the combined extracts thiosulfinate-enriched *Allium sativum* and *Vitis vinifera* after 24 h, 48 h, and 72 h on the Caco-2 cell line.** (**A**) Percentage of cell viability after individual and combined treatments. (**B**) Study of the synergistic effect of the dual combination. * *p*-value of < 0.05 vs. individual treatment. Drug synergy studies were performed with the Combenefit software.

**Figure 6:**
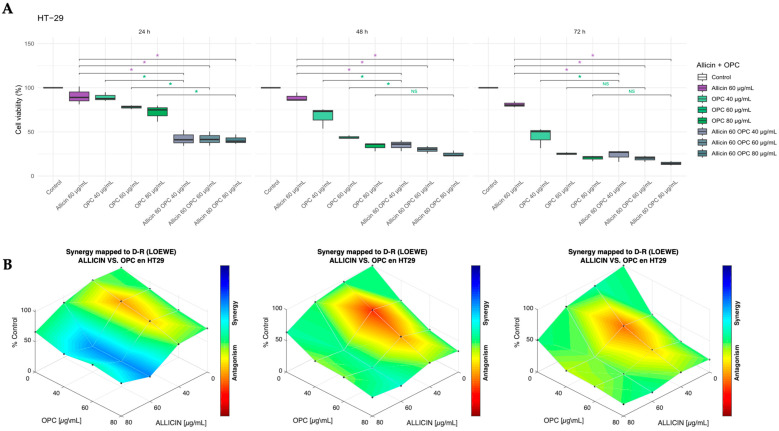
**Effect of the combined extracts thiosulfinate-enriched *Allium sativum* and *Vitis vinifera* after 24 h, 48 h, and 72 h on HT-29 cell line.** (**A**) Percentage of cell viability after individual and combined treatments. (**B**) Study of the synergistic effect of the dual combination. * *p*-value of < 0.05 vs. individual treatment. Drug synergy studies were performed with the Combenefit software.

**Figure 7:**
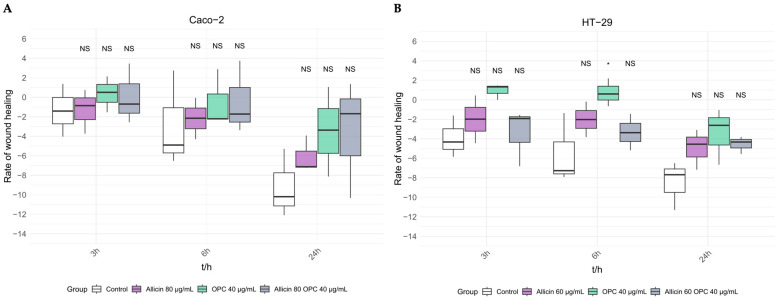
**Cell migration study by scratch wound assay of colon cell lines after 3, 6, and 24 h of treatment with *Allium sativum* and *Vitis vinifera* extracts individually or in combination**. (**A**) Quantification of cell motility in the Caco-2 cell line. (**B**) Quantification of cell motility in the HT-29 cell line * *p*-value of < 0.05 vs. control.

Multiple comparisons against the control group (allicin or OPC treatments) were performed using one-way ANOVA followed by Dunnett’s post hoc test at each time point (Figure 1, Figure 2, Figure 3, Figure 4 and Figure 7). For the combination of extract concentrations between allicin and grape extracts, planned pairwise comparisons were conducted via linear contrasts (multcomp package in R v2024.12.1). Six specific contrasts tested individual extracts (garlic 60/80 µg/mL or grape 40/60/80 µg/mL) against their respective combinations, with *p*-values adjusted via the Holm−Bonferroni method (α = 0.05). Prior to contrasts, one-way ANOVA was applied separately for 24, 48, and 72 h to confirm overall group differences (Figure 5 and Figure 6). Data were processed using the tidyverse and visualized with boxplots showing the distribution of three independent experiments (significance denoted by asterisks). A *p*-value of < 0.05 was considered statistically significant.

## 3. Results

### 3.1. Composition of Vitis vinifera Extract

As mentioned in the Material and Methods Section, we used the allicin and polyphenol content in this work to refer to the dose of *Allium sativum* extract enriched with thiosulfinate and *Vitis vinifera* extract rich in OPCs. The complete list of organic and inorganic compounds contained in the grape derivative used in this study is presented in Table 1.

### 3.2. The Thiosulfinate-Enriched Allium sativum Extract Decreased Viability on Colon Cancer Cells

First, we tested the effect of thiosulfinate-enriched *Allium sativum* extract on the viability of colon cancer cells, Caco-2 and HT-29, as shown in Figure 1 and Figure 2, respectively. Both colon cancer cell lines exhibited a clear reduction in viability, directly proportional to the amount of allicin used and incubation time. In the Caco-2 cell line (Figure 1), a significant reduction in cell viability was observed starting at a concentration of 80 µg/mL compared to the control at 24 h. Higher concentrations (100, 120, and 140 µg/mL) also showed a significant effect, reinforcing the dose-dependent trend. As the exposure time increased to 48 h and 72 h, a progressive decrease in cell viability was evident at the previously mentioned doses.

As seen in Figure 2, the HT-29 cell line, a similar pattern to that observed in Caco-2 cells was noted. At 24 h, a significant reduction in cell viability was detected at 80 µg/mL compared to the control, and higher concentrations (100, 120, and 140 µg/mL) also showed significant effects. This trend remained constant at 48 and 72 h of incubation.

### 3.3. The Vitis vinifera Extract Decreased Viability on Colon Cancer Cells

Second, we tested the effect of *Vitis Vinifera* extract on the viability of Caco-2 and HT-29 cells, as shown in Figure 3 and Figure 4, respectively. A clear reduction in cell viability of both colon cancer lines was observed, directly proportional to the amount of OPCs used and incubation time. The MTT assay revealed a significant decrease in cell viability in Caco-2 cells treated with *Vitis vinifera* extract (from 40 µg/mL OPC) after 24 h of incubation (Figure 3); for HT-29 cells, it was from 60 µg/mL OPC after 24 h of incubation (Figure 4).

### 3.4. Effect of the Combination Thiosulfinate-Enriched Allium sativum Extract with Vitis vinifera Extract Rich in OPCs on Colon Cancer Cell Lines

After that, we explored whether the combination of *Allium sativum* extract enriched with *Vitis vinifera* with high OPCs content for 24 h, 48 h, and 72 h could enhance the effect compared to individual treatments on cell viability of Caco-2 and HT-29 cell lines, as shown in Figure 5 and Figure 6, respectively. In the Caco-2 cell line, we established a pattern of combined administrations (80 µg/mL referred to allicin content), with three different concentrations of *Vitis vinifera* extract (40, 60, and 80 µg/mL); in the HT-29 cell line, the allicin content was 60 µg/mL, and the three different concentrations of the *Vitis vinifera* extract were 40, 60, and 80 µg/mL. As shown in Figure 5A, the combined treatment of Allicin 80 µg/mL with increasing concentrations of *Vitis vinifera* extract (40, 60, and 80 µg/mL) revealed significant time-dependent effects on cell viability in Caco-2 cells, compared to the individual *Allium sativum* extract treatment (indicated by a purple asterisk in Figure 5A). Using the Combenefit software, we determined that the effect of both extracts did not show a synergistic antiproliferative effect on the Caco-2 cell line (Figure 5B).

In HT-29 cells (Figure 6A), on the one hand, all tested combinations of both extracts resulted in a marked decrease in cell viability compared to the individual *Allium sativum* extract treatment (indicated by a purple asterisk in Figure 6A) at all time points (24, 48, and 72 h); on the other hand, when compared to *Vitis vinifera* individual extract, all three combinations led to a significant reduction in cell viability at 24 h. At 48 h, only the two lowest OPC (40 and 60 µg/mL) concentrations maintained this effect, and by 72 h, only the lowest combination, 40 µg/mL OPC, remained significantly different (indicated by the green asterisk in Figure 6A). Thanks to the Combenefit software, it was determined that the effect of both extracts only showed a synergistic antiproliferative effect at 24 h of treatment (Figure 6B).

### 3.5. Antimigratory Effect of Allium sativum and Vitis vinifera Extracts on Colorectal Tumor Lines

In order to test whether *Allium sativum* and *Vitis vinifera* extracts, as well as their combination treatments, exert anti-migration effect on colon cell lines, Caco-2 and Ht-29, a cell scratch wound migration assay was performed (Figure 7A and 7B, respectively). As seen in Figure 7A, we did not observe an anti-migratory effect when treating the Caco-2 cell line with both extracts, either individually or in combination. However, in the HT-29 cell line, OPC treatment at 6 h significantly reduced cell migration, indicating a potential anti-migratory effect. No significant changes were observed for the other conditions (Figure 7B). Representative images of the wound area were obtained at the beginning of the experiment (time 0) and after 3, 6, and 24 h in Caco-2 and HT-29 cells, as shown in Supplementary Figure S1.

## 4. Discussion

Cancer is the second leading cause of death worldwide and represents a major public health challenge, accounting for approximately 10 million deaths annually [15]. Specifically, colorectal cancer is among the most prevalent and lethal malignancies worldwide. Despite advancements in early detection and the development of targeted therapies, CRC remains a substantial health burden due to late-stage diagnosis, chemotherapy resistance, and metastatic progression [16]. Consequently, there is increasing interest in exploring alternative or complementary therapeutic strategies that leverage the potential of natural compounds with anticancer properties. Natural products represent a promising source for the development of novel and effective anticancer drugs due to their low toxicity, structural diversity, and ability to target a wide range of cancers [17].

In a previous work carried out by our experimental group, we explored the use of a new lyophilized garlic extract enhanced through enrichment with thiosulphinate, as an adjuvant in the treatment of colorectal tumors. As a result, it was observed that the combination of freeze-dried garlic extract and 5-FU or oxaliplatin could be presented as a new chemotherapeutic regimen in this pathology [7]. Continuing this line of research, this study presents a new grape extract to explore its potential antitumor effect on colon cancer cells. The extract is obtained from a white grape variety using a specific protocol involving gradient mobile phase HPLC at 28 °C, resulting in a grape extract rich in oligomeric proanthocyanidins ≥ C4. In this new extract, a better efficacy has been achieved in relation to obtaining a higher concentration of OPCs contained in the *Vitis vinifera* extract (Table 1), compared to other studies [18]. According to the scientific literature to date, OPCs inhibit cell proliferation in various types of cancer through mechanisms such as alteration of the cell cycle inducing arrest in G0/G1 and induction of apoptosis [19,20]. In addition, there is no article in the literature that studies the effect of the combination of *Allium sativum* and *Vitis vinifera* extracts on the viability and cell migration in cancer cell lines as a potential adjuvant cancer therapy. All the articles to date study their properties individually in different tumor lineages [21,22,23]. Therefore, we also aim to investigate whether grape extract enhances the efficacy of garlic extract in terms of cell viability and migration.

Firstly, we tested the cytotoxic effects of thiosulfinate-enriched *Allium sativum* and OPC-enriched *Vitis vinifera* extracts in two different human colon cancer cell lines: Caco-2 and HT-29. Our study demonstrates that both extracts significantly reduce the viability of colorectal cancer cell lines in a dose- and time-dependent manner. On the one hand, the allicin concentrations found to significantly decrease colon cancer cell viability (Figure 1 and Figure 2) are in line with the study previously published [7]. On the other hand, the OPC doses that significantly affect cell viability of colorectal tumor lines (Figure 3 and Figure 4) were lower than other studies [24,25].

When evaluating the combination of both extracts, a greater inhibitory effect on cell viability was observed compared to the individual treatments. In the HT-29 cell line, this effect was observed when comparing the combination of both extracts to the individual *Allium sativum* and *Vitis vinifera* extract treatment (Figure 6). However, in the Caco-2 cell line, this effect was observed when comparing only the combination of both extracts to the individual *Allium sativum* extract treatment (Figure 5). The results observed in both Caco-2 and HT-29 cell lines indicate that it is possible that treatment with grape extract alone exerts a significant cell viability inhibitory effect, and therefore, no higher decrease in viability was observed when combining both extracts. Despite the significant effects of dual combination in cell viability, it is interesting to note that a synergistic effect of the extracts was observed in the HT-29 cell line at 24 h (Figure 6), but not at later time points or in the Caco-2 line (Figure 5). This time-limited response suggests that the interaction between these two compounds is not only cell line-specific but also time-dependent. A possible explanation is that the combination may cause an acute increase in reactive oxygen species, leading to amplified apoptotic signaling, mitochondrial dysfunction, and enhanced caspase activation during the early stages of exposure [26,27,28]. Over time, however, cancer cells may activate compensatory survival mechanisms such as upregulation of endogenous antioxidant systems or activation of the Nrf2 pathway, thereby restoring redox homeostasis and reducing apoptosis [29]. Differences in metabolic processing between HT-29 and Caco-2 cells, related to their degree of differentiation, could influence the availability and efficacy of bioactive compounds [30]. Other factors, such as drug stability in the culture medium or the intrinsic capacity of each cell line to adapt to the treatment, may also contribute to the loss of synergy at later time points. This observation highlights the importance of considering both tumor biology and treatment timing when evaluating combinatory strategies, and further molecular studies will be required to confirm these hypotheses.

Although there is extensive literature exploring the role of garlic and grape extracts in cell viability in different types of cancer, studies on the effect of these natural compounds on cell migration, specifically grape extract, are more limited. In concordance to the published literature [18,31], a possible antimigratory effect was observed in HT-29 cells with the individual *Allium sativum* and *Vitis vinifera* extracts treatment, although significance was only reached to the individual *Vitis vinifera* extract treatment (Figure 7B). The combination of the extracts had no significant effect on cell migration. A study conducted by Zhou et al. observed that garlic extract could inhibit the invasiveness of SW480 and SW620 cells, but no effect was observed on the invasive activity of HT-29 cells, which supports our result. They suggested that the anti-invasiveness of the extract might depend on the specific type of cancer cell [32]. The observed discrepancies highlight the need for further studies of this type to help us elucidate the role of these plant extracts in cell migration.

### Limitations

Despite these promising results, the present study has several limitations that should be acknowledged. Although we observed clear effects on cell viability and migration, no molecular analyses were performed to elucidate the specific pathways or molecular targets modulated by either extract in each assay. This lack of mechanistic data limits our ability to draw definitive conclusions about the modes of action involved. Moreover, although the combination of *Allium sativum* and *Vitis vinifera* extracts resulted in a synergistic antiproliferative effect in HT-29 cells, the molecular basis underlying this synergy remains uncharacterized. Future studies will be conducted to elucidate these issues.

## 5. Conclusions

In conclusion, our findings support the therapeutic potential of thiosulfinate-enriched *Allium sativum* and *Vitis vinifera* rich in oligomeric proanthocyanidins extracts in colorectal cancer treatment, particularly in inhibiting cell proliferation. The absence of a sustained synergistic effect and lack of anti-migratory activity highlight the need for further studies to better understand their mechanisms of action and explore strategies to optimize their efficacy in combination with other treatments.

## 6. Patents

Patent WO 2008/102036 A1. Method was used to obtain a freeze-dried, stable extract from plants of the *Allium* genus.

The national patent (Spanish Trademark number: ES2675282A1) covers *Allium sativum* extract, its use for the manufacture of a medicinal product for the treatment of diseases, and its extraction procedure.

## Figures and Tables

**Table 1:** **Composition of Vitis vinifera extract:** compilation of organic and inorganic compounds found in the lyophilized Vitis vinifera extract under optimized conditions. * Oligomeric Proantocyanidins.

Compound	Number of Elementary Units (Ui)	Molecular Weight	Concentration (ppm) HPLC
Gallic Acid	1	170.12	4658.70
B1 *	2	578.52	19,260.47
Epigallocatechin	1	306.27	20,192.92
Catechin	1	290.27	61,204.05
B2 *	2	578.52	33,348.30
Epicatechin	1	290.27	44,091.92
C1 *	3	866.77	7752.06
**OPCs**	**≥4**	**594.5**	**902,491.80**

## Data Availability

The original contributions presented in this study are included in the article. Further inquiries can be directed to the corresponding author.

## Supplementary Materials

The following supporting information can be downloaded at: https://www.mdpi.com/article/10.3390/biomedicines13081968/s1, Figure S1: Representative picture of the area studied by scratch wound assay in Caco-2 and HT-29 cells.

## Abbreviations

The following abbreviations are used in this manuscript:

CRC	colorectal cancer
DMSO	dimethyl sulfoxide
MTT	3-(4,5-dimethylthiazol-2-yl)-2,5-diphenyl-2H-tetrazolium bromide
OPC	oligomeric proanthocyanidins condensed
PA	proanthocyanidins
PBS	phosphate-buffered saline
ROS	reactive oxygen species

## References

[1] Mao Y., Wang W., Yang J., Zhou X., Lu Y., Gao J., Wang X., Wen L., Fu W., Tang F. (2023). Drug repurposing screening and mechanism analysis based on human colorectal cancer organoids. Protein Cell.

[2] Li Q., Geng S., Luo H., Wang W., Mo Y.-Q., Luo Q., Wang L., Song G.-B., Sheng J.-P., Xu B. (2024). Signaling pathways involved in colorectal cancer: Pathogenesis and targeted therapy. Signal Transduct. Target. Ther..

[3] Talib W.H., Baban M.M., Bulbul M.F., Al-Zaidaneen E., Allan A., Al-Rousan E.W., Ahmad R.H.Y., Alshaeri H.K., Alasmari M.M., Law D. (2024). Natural Products and Altered Metabolism in Cancer: Therapeutic Targets and Mechanisms of Action. Int. J. Mol. Sci..

[4] Rana S.V., Pal R., Vaiphei K., Sharma S.K., Ola R.P. (2011). Garlic in health and disease. Nutr. Res. Rev..

[5] Kawasaki H., Nussbaum G. (2025). Therapeutic potential of garlic, aged garlic extract and garlic-derived compounds on pancreatic cancer (Review). Biomed. Rep..

[6] Hoffmann M., Sauer J., Book M., Ermler T.F., Fischer P., Gerlach S., Beltagi K., Morgenroth A., Alexa R., Kranz J. (2025). Mechanism of Action and Interaction of Garlic Extract and Established Therapeutics in Prostate Cancer. Int. J. Mol. Sci..

[7] Perez-Ortiz J.M., Galan-Moya E.M., de la Cruz-Morcillo M.A., Rodriguez J.F., Gracia I., Garcia M.T., Redondo-Calvo F.J. (2020). Cost Effective Use of a Thiosulfinate-Enriched Allium sativum Extract in Combination with Chemotherapy in Colon Cancer. Int. J. Mol. Sci..

[8] Krest I., Keusgen M. (1999). Quality of herbal remedies from Allium sativum: Differences between alliinase from garlic powder and fresh garlic. Planta Med..

[9] Iberl B., Winkler G., Knobloch K. (1990). Products of Allicin Transformation: Ajoenes and Dithiins, Characterization and their Determination by HPLC*. Planta Med..

[10] Shaban N.Z., Hegazy W.A., Abu-Serie M.M., Talaat I.M., Awad O.M., Habashy N.H. (2024). Seedless black *Vitis vinifera* polyphenols suppress hepatocellular carcinoma *in vitro* and *in vivo* by targeting apoptosis, cancer stem cells, and proliferation. Biomed. Pharmacother..

[11] Aldayel T.S., Kilany O.E., El-Hak H.N.G., Abdelrazek H.M.A., Abdallah O., Omar D.E. (2024). Clinicopathological Studies on the Impact of Grape Seed Extract and L-Carnitine as Cardioprotective Agents Against Doxorubicin-Induced Toxicity in Rats. Life.

[12] Wang T.K., Xu S., Li S., Zhang Y. (2020). Proanthocyanidins Should Be a Candidate in the Treatment of Cancer, Cardiovascular Diseases and Lipid Metabolic Disorder. Molecules.

[13] Nie F., Liu L., Cui J., Zhao Y., Zhang D., Zhou D., Wu J., Li B., Wang T., Li M. (2023). Oligomeric Proanthocyanidins: An Updated Review of Their Natural Sources, Synthesis, and Potentials. Antioxidants.

[14] Ravindranathan P., Pasham D., Balaji U., Cardenas J., Gu J., Toden S., Goel A. (2018). A combination of curcumin and oligomeric proanthocyanidins offer superior anti-tumorigenic properties in colorectal cancer. Sci. Rep..

[15] Talib W.H., Baban M.M., Azzam A.O., Issa J.J., Ali A.Y., AlSuwais A.K., Allala S., Al Kury L.T. (2024). Allicin and Cancer Hallmarks. Molecules.

[16] Shin A.E., Giancotti F.G., Rustgi A.K. (2023). Metastatic colorectal cancer: Mechanisms and emerging therapeutics. Trends Pharmacol. Sci..

[17] Choudhari A.S., Mandave P.C., Deshpande M., Ranjekar P., Prakash O. (2020). Phytochemicals in Cancer Treatment: From Preclinical Studies to Clinical Practice. Front. Pharmacol..

[18] Kuhnert S., Lehmann L., Winterhalter P. (2015). Rapid characterisation of grape seed extracts by a novel HPLC method on a diol stationary phase. J. Funct. Foods.

[19] Toden S., Ravindranathan P., Gu J., Cardenas J., Yuchang M., Goel A. (2018). Oligomeric proanthocyanidins (OPCs) target cancer stem-like cells and suppress tumor organoid formation in colorectal cancer. Sci. Rep..

[20] Yang N., Gao J., Cheng X., Hou C., Yang Y., Qiu Y., Xu M., Zhang Y., Huang S. (2017). Grape seed proanthocyanidins inhibit the proliferation, migration and invasion of tongue squamous cell carcinoma cells through suppressing the protein kinase B/nuclear factor-κB signaling pathway. Int. J. Mol. Med..

[21] Pan D., Zheng M., Liu J., Sun Z., Shi X. (2023). Garlic Extract Participates in the Proliferation and Apoptosis of Nonsmall Cell Lung Cancer Cells Via Endoplasmic Reticulum Stress Pathway. Evid. Based Complement. Alternat. Med..

[22] Hu X., Cao B.N., Hu G., He J., Yang D.Q., Wan Y.S. (2002). Attenuation of cell migration and induction of cell death by aged garlic extract in rat sarcoma cells. Int. J. Mol. Med..

[23] Valenzuela M., Bastias L., Montenegro I., Werner E., Madrid A., Godoy P., Párraga M., Villena J. (2018). Autumn Royal and Ribier Grape Juice Extracts Reduced Viability and Metastatic Potential of Colon Cancer Cells. Evid.-Based Complement. Altern. Med. ECAM.

[24] Pérez-Ortiz J.M., Alguacil L.F., Salas E., Hermosín-Gutiérrez I., Gómez-Alonso S., González-Martín C. (2019). Antiproliferative and cytotoxic effects of grape pomace and grape seed extracts on colorectal cancer cell lines. Food Sci. Nutr..

[25] Porcelli L., Iacobazzi R.M., Quatrale A.E., Bergamini C., Denora N., Crupi P., Antonacci D., Mangia A., Simone G., Silvestris N. (2017). Grape seed extracts modify the outcome of oxaliplatin in colon cancer cells by interfering with cellular mechanisms of drug cytotoxicity. Oncotarget.

[26] Zou X., Liang J., Sun J., Hu X., Lei L., Wu D., Liu L. (2016). Allicin sensitizes hepatocellular cancer cells to anti-tumor activity of 5-fluorouracil through ROS-mediated mitochondrial pathway. J. Pharmacol. Sci..

[27] Dinicola S., Mariggiò M.A., Morabito C., Guarnieri S., Cucina A., Pasqualato A., D’Anselmi F., Proietti S., Coluccia P., Bizzarri M. (2013). Grape seed extract triggers apoptosis in Caco-2 human colon cancer cells through reactive oxygen species and calcium increase: Extracellular signal-regulated kinase involvement. Br. J. Nutr..

[28] Sarvizadeh M., Hasanpour O., Naderi Ghale-Noie Z., Mollazadeh S., Rezaei M., Pourghadamyari H., Masoud Khooy M., Aschner M., Khan H., Rezaei N. (2021). Allicin and Digestive System Cancers: From Chemical Structure to Its Therapeutic Opportunities. Front. Oncol..

[29] Liu B., Wang H. (2022). Oxaliplatin induces ferroptosis and oxidative stress in HT29 colorectal cancer cells by inhibiting the Nrf2 signaling pathway. Exp. Ther. Med..

[30] Juan-García A., Fernández-Blanco C., Font G., Ruiz M.J. (2014). Efectos tóxicos de alternariol por ensayos in vitro: Revisión. Rev. Toxicol..

[31] Lv Q., Xia Q., Li J., Wang Z. (2020). Allicin suppresses growth and metastasis of gastric carcinoma: The key role of microRNA-383-5p-mediated inhibition of ERBB4 signaling. Biosci. Biotechnol. Biochem..

[32] Zhou Y., Li X., Luo W., Zhu J., Zhao J., Wang M., Sang L., Chang B., Wang B. (2022). Allicin in Digestive System Cancer: From Biological Effects to Clinical Treatment. Front. Pharmacol..

