# Supplementary material for: Evaluating Allium sativum and Vitis vinifera Extracts as Potential Adjuvant Agents in Colorectal Cancer: Insights from HT-29 and Caco-2 Models
Source: Biomedicines. 2025 Aug 13;13(8):1968. doi: 10.3390/biomedicines13081968 (PMC12383492; doi:10.3390/biomedicines13081968)
Supplement: Supplementary file 1 [file biomedicines-13-01968-s001.zip › biomedicines-3781971-supplementary.pdf]

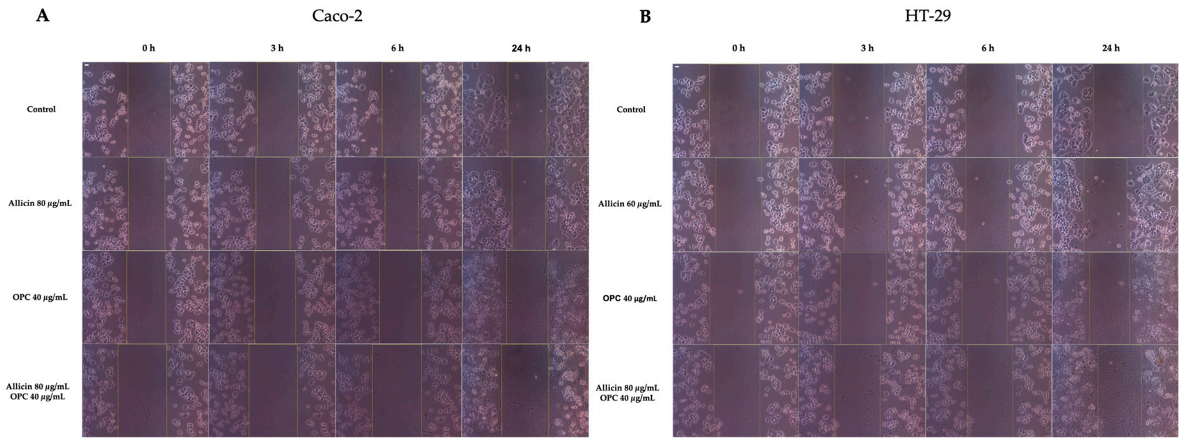

Figure S1. Representative picture of the area studied by scratch wound assay in Caco-2 and HT-29 cells. A: Representative picture of the area studied at the beginning of the experiment (time 0) and after 3 h, 6 h and 24 h in Caco-2 cells; **B:** Representative picture of the area studied at the beginning of the experiment (time 0) and after 3 h, 6 h and 24 h in HT-29 cells). Scale bars: 100μM.
